# Catalytic activities for methanol oxidation on ultrathin CuPt_3_ wavy nanowires with/without smart polymer†
†Electronic supplementary information (ESI) available. See DOI: 10.1039/c6sc01501h


**DOI:** 10.1039/c6sc01501h

**Published:** 2016-05-17

**Authors:** Gengtao Fu, Xiaoxiao Yan, Zhiming Cui, Dongmei Sun, Lin Xu, Yawen Tang, John B. Goodenough, Jong-Min Lee

**Affiliations:** a Jiangsu Key Laboratory of New Power Batteries , Jiangsu Collaborative Innovation Centre of Biomedical Functional Materials , School of Chemistry and Materials Science , Nanjing Normal University , Nanjing 210023 , China . Email: tangyawen@njnu.edu.cn; b Materials Science and Engineering Program & Texas Materials Institute , The University of Texas at Austin , Austin , Texas 78712 , USA; c School of Chemical and Biomedical Engineering , Nanyang Technological University , Singapore 637459 , Singapore . Email: jmlee@ntu.edu.sg

## Abstract

Superior catalytic activity and stability for the methanol oxidation reaction of a direct methanol–air fuel cell is achieved with “clean” ultrathin CuPt_3_ wavy nanowires. The nanowires are synthesized for the first time by functionalizing CuPt_3_ nanoparticles with amine-terminated poly(*N*-isopropylacrylamide), a thermosensitive “smart” polymer having a phase transition at a liquid-electrolyte *T*_t_ (low critical solution temperature, 35 °C). Interestingly, retention of the functionalizing smart polymer on the surface of the nanowires reversibly switches the catalytic activity to lower rates above the *T*_t_. The catalytic performance of the “clean” CuPt_3_ nanowires is shown to be significantly improved over that of commercial Pt black catalyst, owing to their unique structural advantages and bimetallic synergetic effect.

## Abstract 2

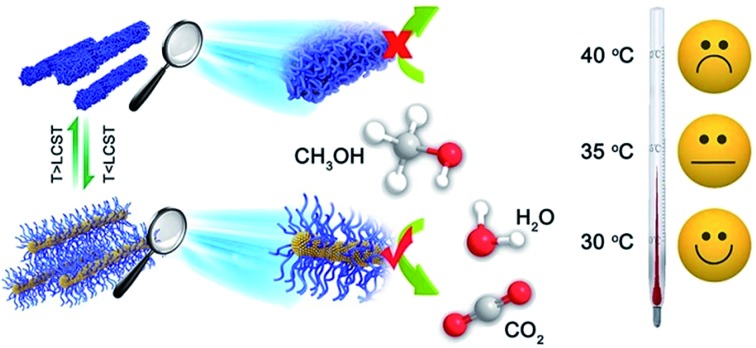
CuPt_3_ nanowires are synthesized with a thermosensitive amine-terminated poly(*N*-isopropylacrylamide) polymer, displaying “smart” temperature-controllable electrocatalytic activity towards the methanol oxidation reaction.

## Introduction

A key component of a direct methanol–air room-temperature fuel cell is an electrochemical catalyst stable over many hours of cell operation for the methanol oxidation reaction (MOR),

1 CH_3_OH + H_2_O → CO_2_ + 6H^+^ + 6e^–^

occurring at the anode. Optimizing the choice of catalyst and the anode morphology is critical to achievement of the required catalytic performance. Since the chemical reaction (1) occurs at the surface of a solid catalyst, these catalysts are synthesized as nanoparticles with a nanosized cross-section in order to create a high surface-to volume ratio.^1^–^10^ An electrocatalyst also needs to be in electronic contact with the electrode current collector as well as with the aqueous electrolyte and the CH_3_OH fuel. Therefore, catalytic nanoparticles are anchored to an electronically conductive substrate with a high surface-to-volume ratio. The substrate normally consists of carbon nanotubes and/or graphene sheets. Alternatively, ultrathin one-dimensional (1D) nanostructures (*e.g.*, ultrathin nanowires and nanorods) represent advantageous geometries,^11^–^16^ having (i) high surface area-to-volume ratio; (ii) high electronic conductivity; (iii) abundant surface defects and (iv) unique quantum effects, all of which endow 1D nanostructures with a high percentage of active atoms exposed on their surface and an improved, high electronic conductivity coefficient as catalysts. The alternative strategy requires a technique to synthesize the nanowires once the catalyst component has been chosen. One synthetic technique consists of coating nanoparticles with a polymer surfactant to keep the particles from aggregating, but the functionalized nanoparticles may bond to one another at open portions of the surface to form a chain of particle segments. The resulting wires are kinked at the particle–particle interface to form wavy nanowires.

Wire-like platinum (Pt) nanostructures have been explored extensively to improve the performance of Pt catalysts.^1^–^7^ Unfortunately, the application of pure Pt catalyst is limited by its relative scarcity and high cost. Alloying Pt with a low-cost metal (M = Cu, Ag, Co, Ni, Fe, Sn, *etc.*) has been an efficient strategy for decreasing Pt utilization and improving catalytic activity.^8^–^18^ For example, PtCu, PtCo, PtNi, PtFe, and PtSnRh wavy nanowires exhibited significantly higher electrocatalytic mass activity towards the methanol oxidation reaction (MOR) than Pt nanowires due to electronic and synergetic effects.^11^,^17^ The catalytic activity of metal nanocrystals is not only structure sensitive but also interface sensitive.^19^–^25^ Surfactants used to avoid nanoparticle aggregation bind to the surface of metallic nanoparticles, thus creating a polymer–metal interface.^26^–^28^ Surfactant polymers that undergo a phase transition in response to an external stimulus (*e.g.* pH, heat, light, an external electric or magnetic field) are referred to as “smart” polymers.^18^,^29^–^36^ They are attracting attention in a variety of fields, including catalysis. A smart polymer coating a catalytic nanoparticle may make the catalytic activity change in response to an external stimulus, particularly where the external stimulus induces a first-order phase change in the polymer.

In this paper, we have used amine-terminated poly(*N*-isopropylacrylamide) (PNIPAM-NH_2_) as a “smart” polymer responsive to temperature in the synthesis of wavy CuPt_3_ nanowires for the first time. The PNIPAM and PNIPAM-NH_2_ molecules are illustrated schematically in Fig. S1a and b.† Similar to the thermosensitive PNIPAM molecules, the PNIPAM-NH_2_ molecules also undergo a first-order phase transition at their solution temperature *T*_t_ (32 °C).^33^,^37^–^40^ They reduce the catalytic activity of the CuPt_3_ nanowires on heating the methanol solution over the *T*_t_. However, the functionalizing polymer can be removed to raise the catalytic performance of the “clean” CuPt_3_ nanowires. Compared to commercial Pt black, the “clean” CuPt_3_ nanowires show a remarkably improved catalytic activity and durability towards the MOR.

## Experimental procedure

### Materials

Amine-terminated poly(*N*-isopropylacrylamide) (PNIPAM-NH_2_) was purchased from Sigma-Aldrich trading Co., Ltd (Shanghai, China). Chloroplatinic acid hexahydrate (H_2_PtCl_6_·6H_2_O) and cupric chloride (CuCl_2_) were purchased from Sinopharm Chemical Reagent Co., Ltd (Shanghai, China). Commercial Pt black was purchased from Johnson Matthey Chemicals Ltd (Shanghai, China). All the reagents were analytical grade and used as received without further purification.

### Synthesis and characterization of the CuPt_3_ wavy nanowires

In a typical synthesis, 0.25 mL of CuCl_2_ (50 mM), 0.75 mL of H_2_PtCl_6_ (50 mM) and 1.0 mL of PNIPAM-NH_2_ (50 mM) as aqueous solutions were added into 7.0 mL of water, followed by continuous stirring for 10 min. The mixed solution was transferred to a 20 mL Teflon-lined stainless-steel autoclave. The autoclave was then heated at 140 °C for 6 h before being cooled down to room temperature. The products were separated by high-speed centrifugation, washed three times with ethanol to remove the excess surfactants, and then dried in an oven for further characterization and application.

Transmission electron microscopy (TEM), high-resolution TEM (HRTEM), high-angle annular dark-field scanning transmission electron microscopy (HAADF-STEM), and energy dispersive X-ray (EDX) elemental mapping measurements were carried out with a JEOL JEM-2100F transmission electron microscope operated at an accelerating voltage of 200 kV. The samples were prepared by placing a drop of the colloidal solution or catalyst powder dispersion in ethanol solution (99%) on a carbon film-coated Ni grid (3 mm, 300 mesh), followed by drying under ambient conditions (25 °C). Scanning electron microscopy (SEM) images were obtained on a JSM-2010 microscope at an accelerating voltage of 20 kV. The crystallinity of the samples was determined by recording X-ray diffraction (XRD) on a Model D/max-rC X-ray diffractometer using a Cu Kα radiation source (*λ* = 1.5406 Å) and operating at 40 kV and 100 mA. X-ray photoelectron spectroscopy (XPS) measurements were performed with a Thermo VG Scientific ESCALAB 250 spectrometer with a monochromatic Al Kα X-ray source. The electron binding energy was calibrated by means of the C 1s peak energy of 284.6 eV. Fourier-transform infrared (FT-IR) spectroscopy was carried out with a Nicolet 520 SXFTIR spectrometer. The thermosensitive property was measured with a dynamic light scattering (DLS) analyser (Malvern Zetasizer Nano ZS90). All ICP-AES measurements were carried out on an IRIS Intrepid instrument (Thermo Fisher, USA). An automatic controlled electric heating digestion system (AAnalyst 400, PerkinElmer, USA) was used for sample digestion.

### Electrochemical measurements

Methanol oxidation experiments were carried out with a CHI 760C electrochemical analyzer (Shanghai, Chenghua Co.). A conventional three-electrode system was used, including a glassy carbon electrode with 3 mm diameter as the working electrode, a Pt wire as the auxiliary electrode, and a saturated calomel reference electrode (SCE). All electrode potentials were quoted *versus* the SCE. Typically, 10 mg of the electrocatalyst sample was sonicated in 5 mL of H_2_O for 30 min to form a homogeneous catalyst ink. Then, 12 µL of the resulting suspension was drop-cast onto the surface of the glassy carbon electrode. After drying at room temperature, the modified electrode surface was covered with 3 µL of Nafion solution (5 wt%) and allowed to dry to obtain the working electrode. The methanol oxidation reaction was performed in N_2_-saturated 0.5 M H_2_SO_4_ + 0.5 M CH_3_OH solution.

## Results and discussion

### Characterization of the smart CuPt_3_ wavy nanowires

**Fig. 1:**
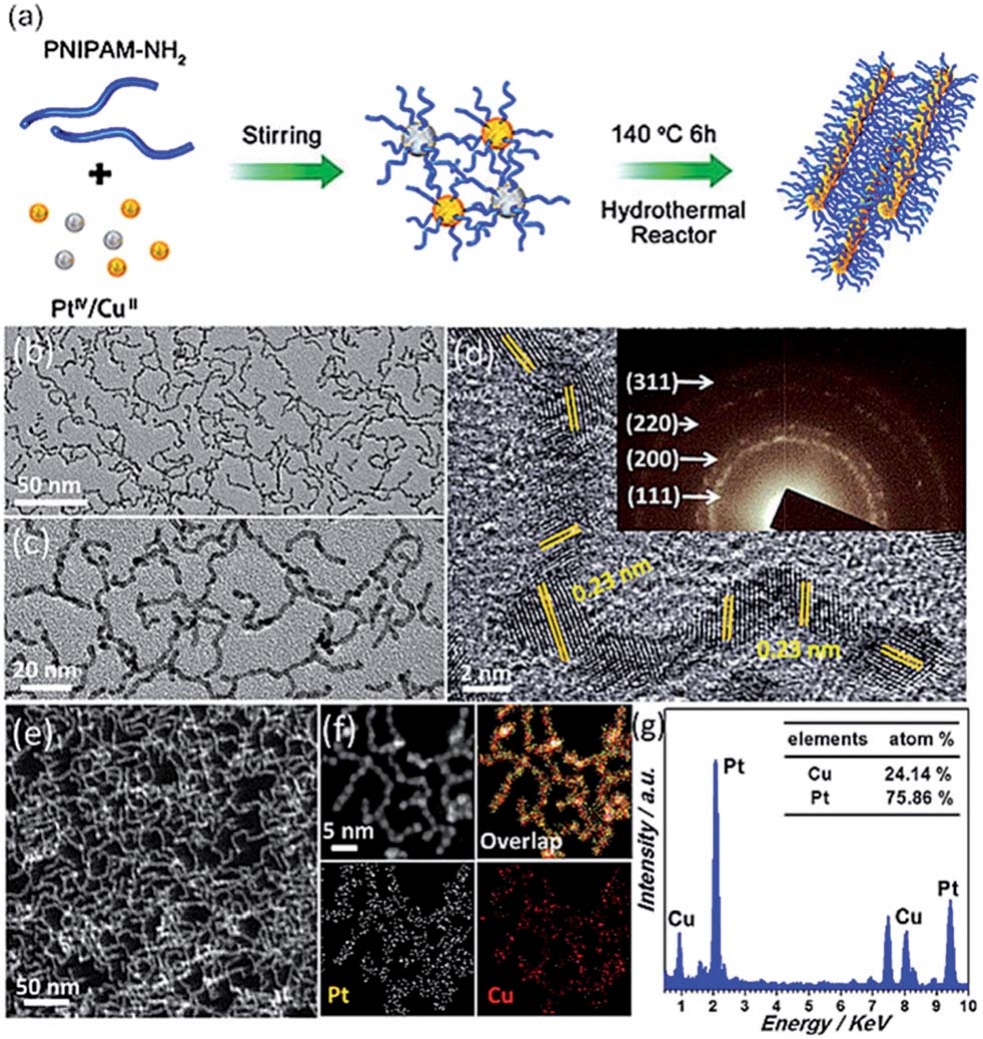
(a) Schematic illustration of the synthesis of PNIPAM-NH_2_-functionalized CuPt_3_ wavy nanowires. Morphological and structural characterization: (b) typical TEM image, (c) magnified TEM image, (d) HRTEM image, (e) HAADF-STEM image, (f) EDX elemental mapping image and (g) EDX spectrum of the ultrathin CuPt_3_ wavy nanowires. The inset in (d) shows the corresponding SAED pattern.

To prepare smart CuPt_3_ wavy nanowires, H_2_PtCl_6_·6H_2_O, CuCl_2_ and PNIPAM-NH_2_ were dissolved in deionized water under magnetic stirring for around 10 min, as shown in Fig. 1a. The resulting homogeneous mixture was transferred to a Teflon-lined stainless-steel autoclave and then heated at 140 °C for 6 h before being cooled to room temperature. The resulting colloidal nanocrystals were collected by centrifugation and washed three times with an ethanol solution (see Experimental section for details).

The morphology and structure of the resulting products were initially characterized by electron microscopy. Typical TEM images (Fig. 1b and c) confirm that abundant wavy nanowires are the dominant products, with high uniformity in terms of the morphology (*ca.* 100%) and ultrathin diameter (*ca.* 2.0 nm). All the wavy nanowires are frequently bent on the TEM grid, indicating their highly flexible nature.^8^,^12^ Fig. 1d shows the HRTEM image of the wavy nanowires. The lattice fringes are not continuous and their orientations vary due to the highly wavy features,^8^ confirming the polycrystalline nature of the wavy nanowires, as demonstrated by the corresponding SAED pattern (Fig. 1d, inset). The lattice spacing of each parallel fringe was calculated to be *ca.* 0.23 nm, corresponding to the {111} facets of face-centered cubic (fcc) Pt/Cu. A deeper observation reveals that the wavy nanowires are fused together closely in the joint region. The wavy wire-like nanostructures are also clearly seen in the HAADF-STEM image (Fig. 1f) and SEM image (Fig. S2†). The elemental distribution of the CuPt_3_ wavy nanowires was investigated by EDX elemental mapping. As seen in Fig. 1e, both Pt and Cu elements are uniformly distributed throughout the nanostructures, confirming the homogeneous alloyed structure of the wavy nanowires. The Cu/Pt atomic ratio was determined to be 24.14 : 75.86 with a TEM equipped with a EDX spectrometer (Fig. 1g), in accordance with the theoretical 25 : 75 stoichiometric proportion.

**Fig. 2:**
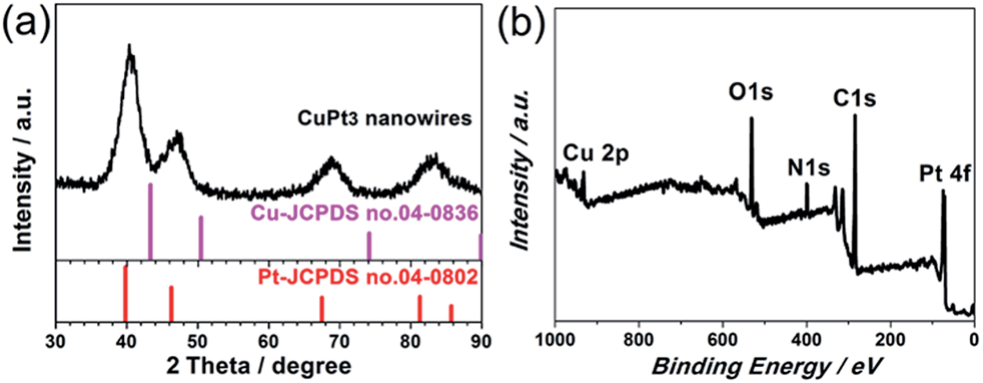
(a) XRD pattern and (b) XPS survey scan spectrum of the CuPt_3_ wavy nanowires.

The XRD diffraction peaks for the CuPt_3_ wavy nanowires are positioned between pure fcc Pt (JCPDS no. 04-0802) and Cu (JCPDS no. 04-0836) crystal phases. No peaks associated with pure Pt or Cu are observed, suggesting the formation of a CuPt_3_ alloy with high purity (Fig. 2a). The broadened diffraction peaks reflect the small dimension of the nanocrystals, further confirming the ultrathin feature of these wavy nanowires. The surface chemical composition of the CuPt_3_ wavy nanowires was further investigated by XPS. The XPS survey scan spectrum reveals that the surface Cu/Pt atomic ratio is *ca.* 24.40 : 75.60 (Fig. 2b), indicating that the same composition is present at the surface as in the bulk. Detailed XPS spectra show that the Pt 4f binding energies (BEs) of the CuPt_3_ wavy nanowires shift to higher BEs with respect to those of bulk Pt, while the Cu 2p BEs negatively shift compared to those of bulk Cu (Fig. S3†), which is indicative of a change in the electronic structure of Pt upon alloying with Cu.^17^

**Fig. 3:**
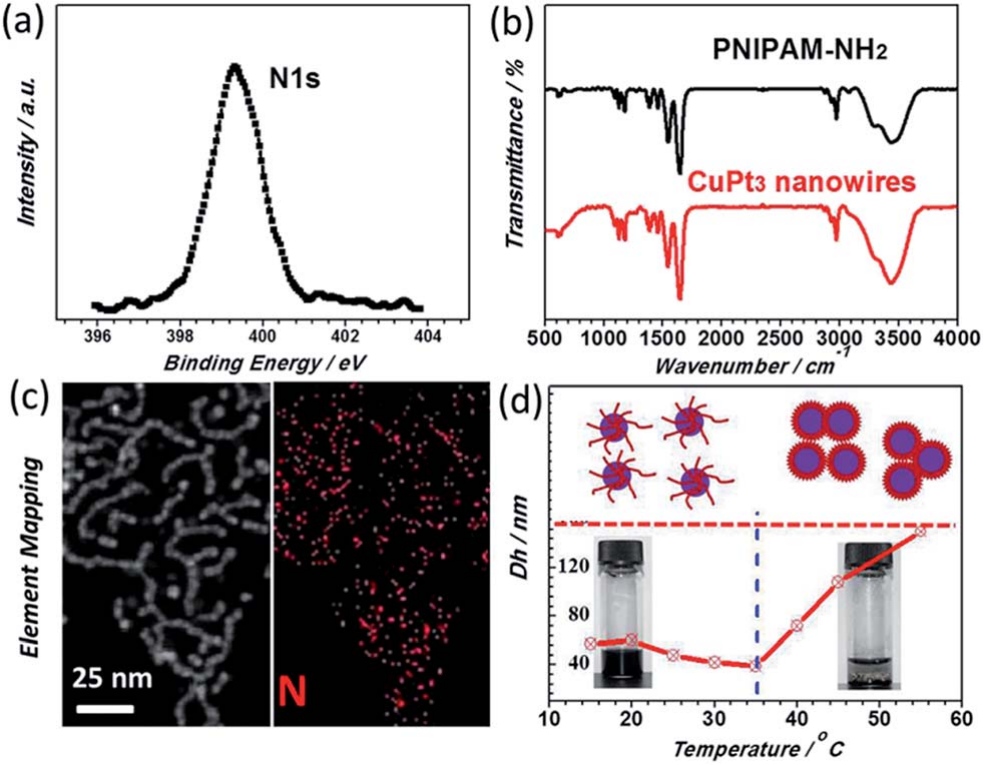
(a) N 1s XPS spectrum of the CuPt_3_ wavy nanowires. (b) FT-IR spectra of the CuPt_3_ wavy nanowires and pure PNIPAM-NH_2_. (c) EDX mapping of the N element. (d) Hydrodynamic diameter of the CuPt_3_ wavy nanowires and pure PNIPAM-NH_2_ as a function of temperature; top panel: a schematic illustrating the phase transition process of the CuPt_3_ wavy nanowires. Bottom panel: the digital images of the CuPt_3_ wavy nanowires at different temperatures in aqueous solution.

In particular, a N 1s XPS peak at 399.3 eV is detected (Fig. 3a), indicating that PNIPAM-NH_2_ is definitely located at the surface of the CuPt_3_ wavy nanowires. This result was further confirmed by the FT-IR spectrum and EDX elemental mapping. It is observed that the FT-IR spectrum of the CuPt_3_ wavy nanowires is similar to that of pure PNIPAM-NH_2_ (Fig. 3b) and the N element mapping pattern is uniformly distributed across the entirety of the wavy nanowires (Fig. 3c). Previous studies have shown that the metal ions could interact strongly with the N atoms of the PNIPAM-NH_2_ by complexation,^41^ which might be responsible for a chemisorption of PNIPAM-NH_2_ on the nanowire surface to form a PNIPAM-NH_2_ functional layer. Fig. 3d presents the temperature dependent hydrodynamic diameters of the PNIPAM-NH_2_-functionalized CuPt_3_ nanocrystals and pure PNIPAM-NH_2_, analyzed by dynamic light scattering (DLS). Similar to pure PNIPAM-NH_2_, the PNIPAM-NH_2_-functionalized CuPt_3_ nanocrystals exhibit a typical thermo-sensitivity. However, the *T*_t_ of the CuPt_3_ wavy nanowires is about 35 °C, slightly higher than that of pure PNIPAM-NH_2_ (32 °C), which may be attributed to the interaction between CuPt_3_ and PNIPAM-NH_2_. Below the *T*_t_ (15–35 °C), the hydrodynamic diameter (*D*_h_) decreases slightly from 57 nm to 39 nm. Above the *T*_t_ (35–55 °C), the *D*_h_ starts to increase dramatically from 39 nm to 150 nm, which is indicative of aggregation of nanocrystals (Fig. S4†). As expected, this result suggests the switchable properties of the CuPt_3_ wavy nanowires may be achieved by using temperature as a trigger.

**Fig. 4:**
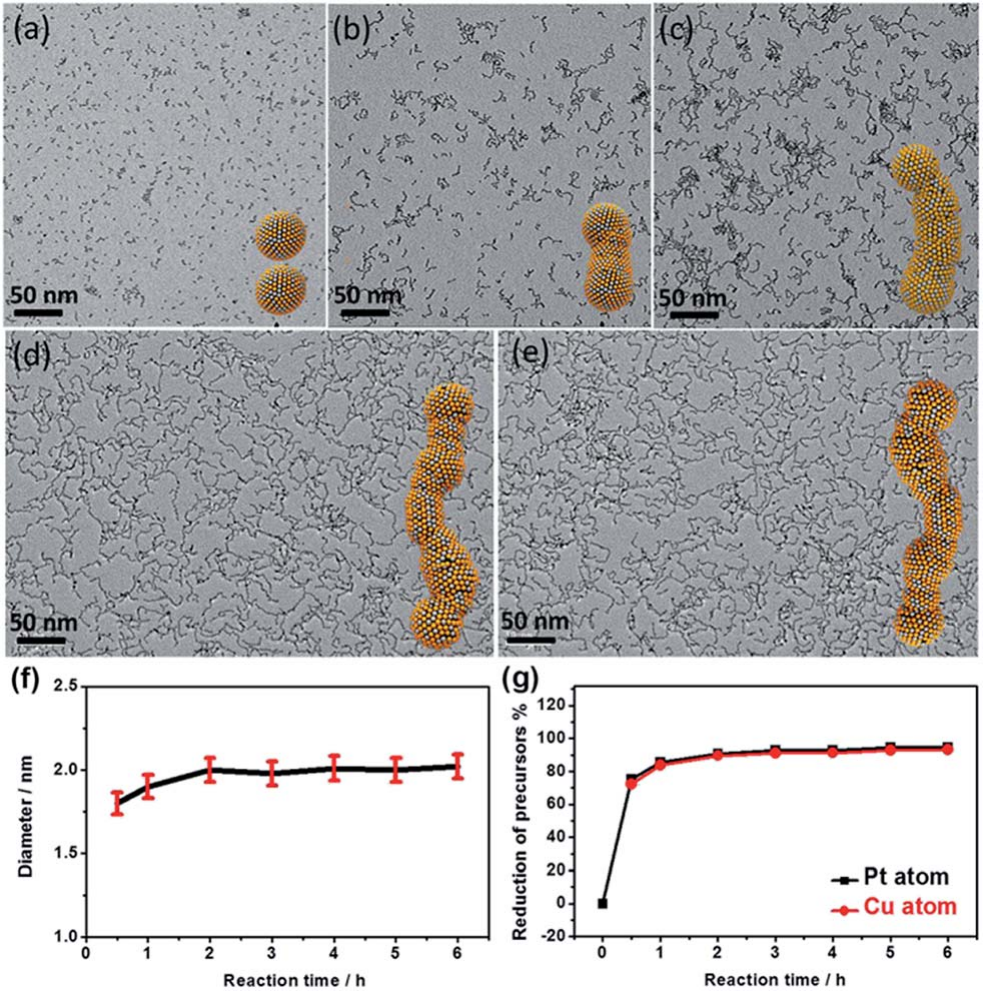
TEM images and the corresponding structure models of the CuPt_3_ wavy nanowires collected at different growth stages: (a) 0.5 h, (b) 1 h, (c) 2 h, (d) 4 h and (e) 6 h. (f) The diameter distribution of the nanowires as a function of reaction time. (g) Data of reduction in H_2_PtCl_6_ and CuCl_2_ during the reaction, calculated from the ICP-AES measurements.

Control experiments show that the wavy nanowires could only be obtained with a sufficient amount of PNIPAM-NH_2_. It is noted that the products are dominated by a mixture of CuPt_3_ nanowires and isolated nanospheres if the amount of PNIPAM-NH_2_ is insufficient (*e.g.*, 0.5 mL), as confirmed by the TEM image (Fig. S5a†), XRD and EDX spectra (Fig. S6†). With a sufficient or excessive PNIPAM-NH_2_ content (1 mL and 2 mL), well-defined wavy nanowires were observed (Fig. S5b and c†). These results illustrate that the PNIPAM-NH_2_ molecules act as a structure-directing agent to induce the growth of the wavy nanowires by the oriented-attachment growth mechanism.^1^,^11^,^17^ The oriented-attachment growth mechanism was further confirmed by a time-dependent evolution experiment (Fig. 4 and S7†). This clearly shows that the morphologies of the CuPt_3_ nanocrystals evolve from isolated nanoparticles to nanowires. The width of the wavy nanowires is largely determined by the nanoparticle diameter formed in the initial stage (Fig. 4f). The composition changes of the CuPt_3_ wavy nanowires were further investigated by using inductively coupled plasma atomic emission spectrometry (ICP-AES). Fig. 4g shows the decrease in H_2_PtCl_6_ and CuCl_2_ as a function of time. As observed, the reduction in H_2_PtCl_6_ and CuCl_2_ is very fast within 30 min, and almost complete within 60 min. No significant change in the overall composition is observed in products beyond 1 h.

Based on these experimental observations, three distinct growth stages can be expressed as follows: (i) in the initial reaction stage (within 30 min), only separated particles were found, with an average size of 1.8 nm, which were likely formed through continuous reduction of the precursors and subsequent growth of the initial crystal nucleus. (ii) With the depletion of the precursors after 60 min, the growth of particles stopped and the particle attachment began to occur. (iii) With the increase in reaction time, their wavy feature became more and more prominent with increasing length of the nanowires. During the process of oriented-attachment, the Cu/Pt atoms near the joints can go through an Oswald ripening process and diffuse along the nanowire due to the relatively low energy barrier, resulting in a relatively smooth interface with the high synthesis temperature.^42^,^43^

Previous results have suggested that the oriented attachment is most likely due to the adsorption of the capping agent to specific facets.^11^,^44^In the current work, the oriented attachment of CuPt_3_ wavy nanowires is likely caused by the preferential adsorption of PNIPAM-NH_2_ on {100} or {110} facets, leading to the growth of CuPt_3_ wavy nanowires along the wavy nanowires along the 〈111〉 direction. It is worthwhile noting that wavy nanowires with a similar diameter of around 2.0 nm can also be obtained by simply tuning the molar ratio of H111 wavy nanowires along the 〈111〉 direction. It is worthwhile noting that wavy nanowires with a similar diameter of around 2.0 nm can also be obtained by simply tuning the molar ratio of H direction. It is worthwhile noting that wavy nanowires with a similar diameter of around 2.0 nm can also be obtained by simply tuning the molar ratio of H_2_PtCl_6_ to CuCl_2_ in the reaction system, as confirmed by the TEM images (Fig. S8a and b†). The corresponding XRD results of the wavy nanowires are given in Fig. S8c.† It can be seen that the peaks shift to higher angles, and no signals associated with pure Pt or Cu are observed, indicating that these PtCu wavy nanowires are also alloy structures. The composition of these wavy nanowires was characterized by EDX (Fig. S8d†). Based on the analysis of EDX, Cu_24.95_Pt_75.05_ and Cu_26.38_Pt_73.62_ were obtained when the molar ratios of CuCl_2_/H_2_PtCl_6_ were 1/1 and 3/1, respectively. It is very clear that the feeding ratios of the Cu and Pt precursors had very limited influence on the morphology and compositions of the CuPt_3_ wavy nanowires, most likely due to the difficulty in reducing Cu^2+^/Cu and the strong tendency of Cu to alloy with Pt to form CuPt_3_ alloy.^45^

### MOR activities on CuPt_3_ wavy nanowires with smart polymer

**Fig. 5:**
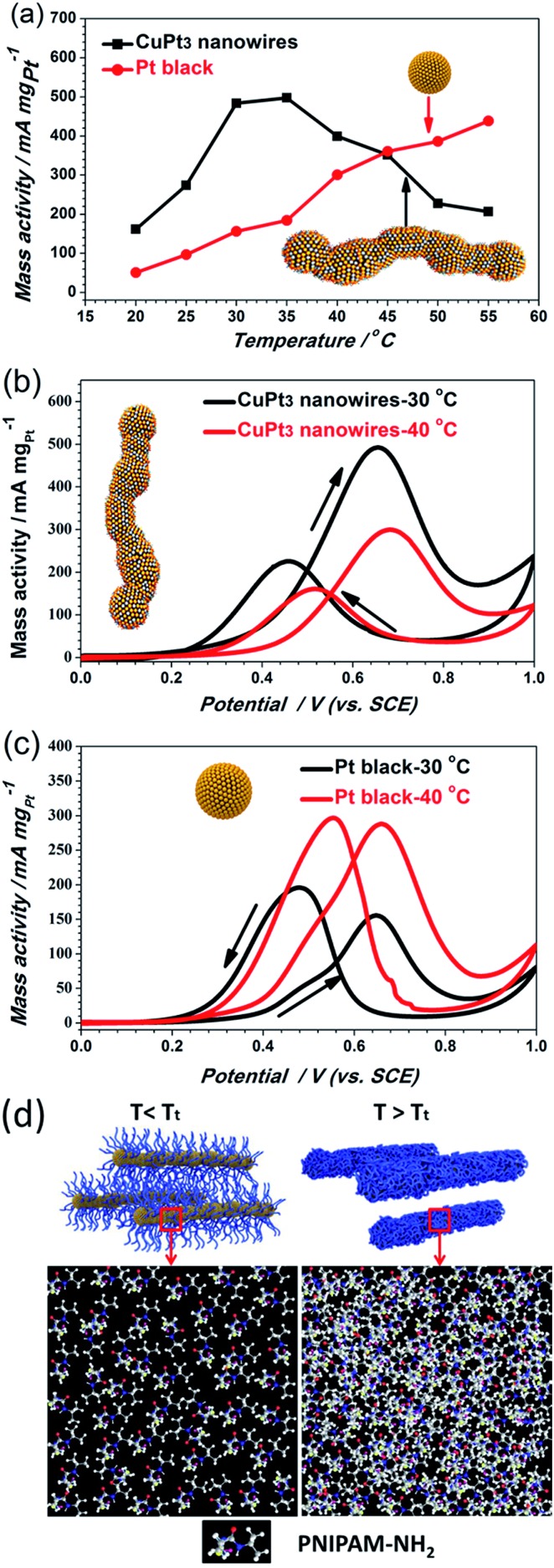
(a) Data of the electrocatalytic activities of the CuPt_3_ wavy nanowires and Pt black towards the MOR as a function of temperature. CVs of the MOR for the (b) CuPt_3_ wavy nanowires and (c) Pt black recorded at two representative temperatures: 30 °C and 40 °C. (d) Schematic illustrations of possible transformations of PNIPAM-NH_2_ on CuPt_3_ surfaces at temperatures below and above the *T*_t_ (note: all electrochemical data recorded in N_2_-saturated 0.5 M CH_3_OH + 0.5 M H_2_SO_4_ solution at a scan rate of 50 mV s^–1^).

Although the CuPt_3_ wavy nanowire surface is covered by PNIPAM-NH_2_, the electrochemical surface area (ECSA) of the CuPt_3_ wavy nanowires (20.3 m^2^ g^–1^) is slightly larger than that of Pt black catalyst (18.0 m^2^ g^–1^), as calculated based on the cyclic voltammograms (CVs) in a N_2_-saturated 0.5 M H_2_SO_4_ solution at 30 °C (Fig. S9a†). However, at 40 °C (*T* > *T*_t_), the ECSA of the CuPt_3_ wavy nanowires is dramatically decreased to a much lower level (Fig. S9b†). This result hints that a temperature-responsive electrocatalyst has been successfully prepared. Subsequently, the MOR activity of the CuPt_3_ wavy nanowires was measured at different temperatures in a N_2_-saturated 0.5 M CH_3_OH + 0.5 M H_2_SO_4_ solution (Fig. S10 and 5b). To eliminate the influence of temperature on the electrocatalytic activity, Pt black was used as a benchmark for comparison (Fig. S11 and 5c). Generally, the typical CVs of the MOR have two obvious oxidation peaks: the oxidation peak in the forward scan originates from methanol oxidation, while the backward scan oxidation peak is associated with the removable CO, which can act as a poisoning species.^46^,^47^ Fig. 5a shows the plots of the oxidation peak current density as a function of temperature. As observed, the CuPt_3_ wavy nanowires showed a sharply different thermosensitive behavior towards the MOR at temperatures below and above the *T*_t_ = 35 °C. For example, at 30 °C (*T* < *T*_t_), the peak current density of mass activity (489.37 mA mg^–1^) on the CuPt_3_ wavy nanowires is 3.04 times larger than that at 40 °C (*T* > *T*_t_) (159.28 mA mg^–1^, Fig. 5b).

However, the electrocatalytic activities on Pt black increased significantly with increased temperature (Fig. 5a and c). This result manifests again that the sharp difference in electrocatalytic activities of the CuPt_3_ wavy nanowires at temperatures below and above *T*_t_ can be attributed to the thermosensitive behaviour of PNIPAM-NH_2_. Below the *T*_t_, the PNIPAM-NH_2_ chains adopt an extended conformation, allowing reagents to easily diffuse through the polymer coating to the CuPt_3_ active surface (Fig. 5d). Above the *T*_t_, the PNIPAM chains collapse to form a thick layer covering the catalyst surface, which results in a decrease in diffusion of reactants to the CuPt_3_ active sites (Fig. 5d). It is concluded that the PNIPAM-NH_2_-functionalized CuPt_3_ wavy nanowires can exhibit controlled electrocatalytic activity by using temperature as a trigger.

### MOR activities on CuPt_3_ wavy nanowires without smart polymer

**Fig. 6:**
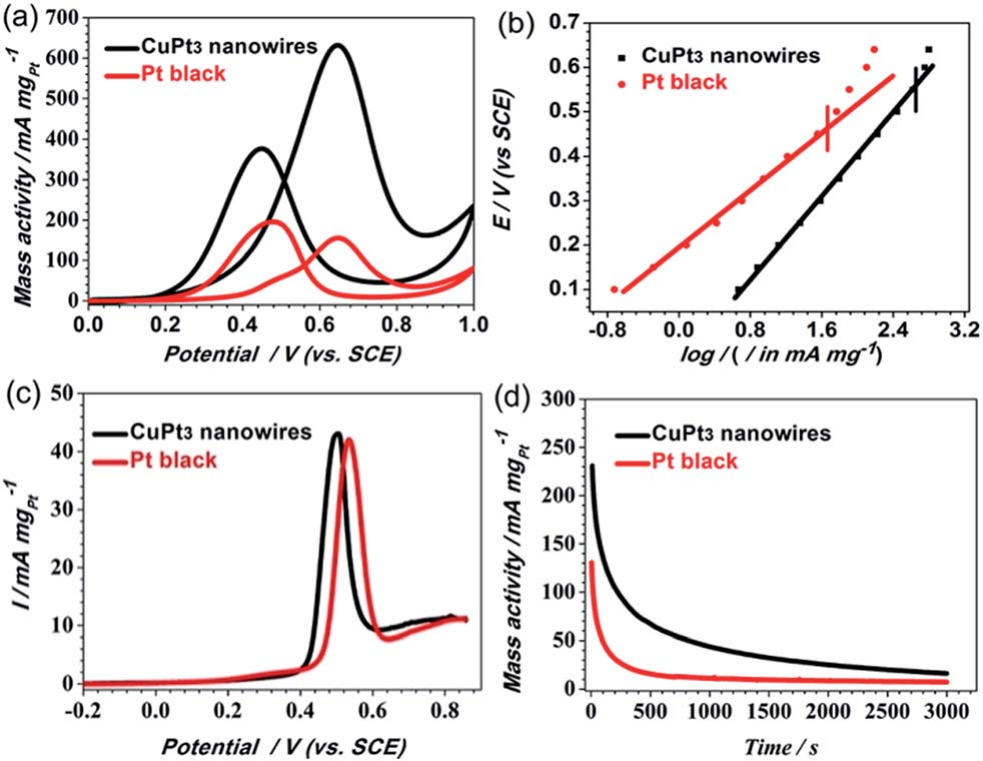
(a) Pt mass-normalized CVs for the CuPt_3_ wavy nanowires and Pt black recorded at 30 °C. (b) Tafel plots of log *I vs. E* for the MOR at CuPt_3_ wavy nanowires and Pt black in electrochemical control area. (c) Representative CO stripping LSV curves for the CuPt_3_ wavy nanowires and Pt black recorded at 30 °C. (d) Chronoamperometry curves for the CuPt_3_ wavy nanowires and Pt black recorded at 30 °C (note: all recorded in N_2_-saturated 0.5 M CH_3_OH + 0.5 M H_2_SO_4_ solution at a scan rate of 50 mV s^–1^).

We believe that ultrafine 1D nanowires represent an ideal structure due to their large surface-to-bulk ratio and their abundant active sites along the longitudinal direction of the nanowires. We believe that ultrafine 1D nanowires represent an ideal structure due to their large surface-to-bulk ratio and their abundant active sites along the longitudinal direction of the nanowires.^11^,^16^,^44^ Thus, the ultrathin CuPt_3_ wavy nanowires may be advantageous as an electrocatalyst. To confirm this conjecture, we compared the electrocatalytic activities of the CuPt_3_ wavy nanowires and Pt black. Before the electrochemical test, the CuPt_3_ wavy nanowires were treated with UV/ozone irradiation (wavelength at 185 nm and 254 nm) for 4 h in air to remove the PNIPAM-NH_2_ covering the nanowire surface. After the UV/Ozone cleaning, the N 1s peak cannot be observed clearly (Fig. S12†), indicating that the PNIPAM-NH_2_ on the CuPt_3_ surface is effectively removed. Based on the high-resolution Pt 4f and Cu 2p XPS spectra (Fig. S13†), the percentages of Pt^2+^ and Cu^2+^ species are calculated to be 16.4% and 38.0%, respectively, which are slightly larger than those of the functionalized CuPt_3_ nanowires (Fig. S3,† Pt^2+^: 15.2%; Cu^2+^: 32.0%), owing to the oxidation by the UV–ozone. The electrocatalytic activity of the “clean” CuPt_3_ wavy nanowires was clearly enhanced compared with that of the untreated CuPt_3_ wavy nanowires (Fig. S14†). Fig. 6a shows the mass-normalized CVs for the MOR on the CuPt_3_ wavy nanowires and Pt black. Noticeably, the ratio of the forward anodic peak current (*I*_f_) to the backward anodic peak current (*I*_b_) on the CuPt_3_ wavy nanowires (*I*_f_/*I*_b_ = 1.67) is much higher than that on Pt black (*I*_f_/*I*_b_ = 0.79), revealing that the MOR on the CuPt_3_ wavy nanowires is mainly by a direct pathway (*i.e.*, good CO tolerance). From the Tafel plots of the two catalysts displayed in Fig. 6b, it is observed that the two obtained plots exhibit a good linear relationship in the lower current region. In comparison with Pt black, the CuPt_3_ wavy nanowires show a higher output current under the same potentials. Moreover, the polarization over-potential at the CuPt_3_ wavy nanowires appears at a relatively higher output current density. These results suggest that the MOR occurring at the CuPt_3_ wavy nanowires can markedly reduce the overpotential and has a faster kinetic rate. The improved CO tolerance of the CuPt_3_ wavy nanowires was further confirmed by CO-stripping linear sweep voltammograms (LSVs). As observed in Fig. 6c, the CuPt_3_ wavy nanowires show clear negative shifts in the onset and peak potentials for the MOR relative to the Pt black catalyst, indicating that the CuPt_3_ wavy nanowires are more active for CO oxidation than Pt black. In addition, the CuPt_3_ wavy nanowires exhibited an enhanced mass activity (Fig. 6a) and specific activity (Fig. S15†) compared to those of Pt black. For example, the peak current densities of mass activity (634.78 mA mg^–1^) and specific activity (2.80 mA cm^–2^) on the CuPt_3_ wavy nanowires are 3.90 and 3.11 times larger than those on Pt black (mass activity: 162.54 mA mg^–1^ and specific activity: 0.90 mA cm^–2^). It is worthwhile to note that the specific activity at the CuPt_3_ wavy nanowires towards the MOR is also higher than that at other PtCu catalysts, such as Pt_1_Cu_2_ nanowires (1.87 mA cm^–2^),^17^ Pt_3_Cu_1_ icosahedra (2.14 mA cm^–2^),^45^ Pt_3_Cu_1_ pyramid caps (*ca.* 2.10 mA cm^–2^),^48^ and hollow Pt_1_Cu_1_ (2.08 mA cm^–2^).^49^ Given that the electrocatalytic stability is important for an electrocatalyst, we performed chronoamperometry (CA) measurements in 0.5 M H_2_SO_4_ + 0.5 M CH_3_OH solution at a fixed potential of 0.6 V. As shown in Fig. 6d, the polarization currents for both catalysts decrease rapidly because of the formation of CO species during the MOR process.^17^,^50^ Compared with Pt black, the CuPt_3_ wavy nanowires show a slower current density decay over time, indicating the better CO tolerance of the Pt–Cu wavy nanowires, which is also consistent with the above CV results.

The enhanced electrocatalytic performance of the wavy CuPt_3_ nanowires can be attributed to the following aspects. First, the ultrathin wavy nanowires provide many unique structural advantages: (i) the ultrathin nanowires possess more exposed active sites because of their high surface area-to-volume ratio; (ii) distinct defects such as atomic steps and crystal face defects are frequently observed around the vicinity of the crystal domains of the nanowire (Fig. S16†), which may create more surface active sites and serve as possible channels for O_2_ incorporation into the surface region, thus potentially contributing to the oxidation process;^8^,^12^,^42^ (iii) wavy wire-like structures are favourable for electron transport and mass transfer of the reactants and resultants, thus promoting the kinetic processes of the MOR; the electronic structure change of surface Pt is another important factor for improving the catalytic activity. The alloying of Cu with Pt changes the electron binding energy on Pt, as verified by XPS, which is beneficial for C–H cleavage at low potential and weakens the chemisorption of CO poisonous intermediates formed on the CuPt_3_ surfaces.^17^,^18^ Based on the above results, it is concluded that the improved electrocatalytic activity and stability of the CuPt_3_ wavy nanowires may be attributed to their unique structural advantages and the synergistic effect between Pt and Cu.

## Conclusions

In summary, the ultrathin CuPt_3_ wavy nanowires show superior activity and durability for the MOR of a direct methanol–air fuel cell. The nanowires consist of a chain of bonded nanoparticles, afforded by coating the particles with the polymer PNIPAM-NH_2_. The alloyed CuPt_3_ structure and the morphology of the “clean” wavy nanowires enhance the performance of the nanoparticles relative to that of Pt black catalyst. In addition, PNIPAM-NH_2_ is a thermosensitive smart polymer, which provides a switching of the activity of the CuPt_3_ wavy nanowires that retain the polymer coating by heating across a first-order polymer phase change at *T*_t_ (35 °C). This result shows that the ultrathin CuPt_3_ wavy nanowires not only can be utilized as a promising anodic electrocatalyst in direct methanol–air fuel cells, but may also pave the way for designing temperature-switchable nanoreactors.

## Supplementary Material

Supplementary information
