# Supplementary material for: Catalytic activities for methanol oxidation on ultrathin CuPt3 wavy nanowires with/without smart polymer
Source: Chem Sci. 2016 May 17;7(8):5414–20. doi: 10.1039/c6sc01501h (PMC6054026; doi:10.1039/c6sc01501h)
Supplement: Supplementary file 1 [file SC-007-C6SC01501H-s001.pdf]

Electronic Supplementary Material (ESI) for Chemical Science.
This journal is © The Royal Society of Chemistry 2016

Supporting information

Catalytic activities for methanol oxidation on ultrathin $CuPt_3$ wavy nanowires with/without smart polymer

Gengtao Fu,[a,b,‡] Xiaoxiao Yan,[a,‡] Zhiming Cui,[b] Dongmei Sun,[a] Lin Xu,[a,c] Yawen Tang,[a,*] John B. Goodenough[b] and Jong Min Lee[c,*]

[a] Jiangsu Key Laboratory of New Power Batteries, Jiangsu Collaborative Innovation Centre of Biomedical Functional Materials, School of Chemistry and Materials Science, Nanjing Normal University, Nanjing 210023, China

[b] Materials Science and Engineering Program & Texas Materials Institute, The University of Texas at Austin, Austin, Texas 78712, United States

[c] School of Chemical and Biomedical Engineering, Nanyang Technological University, Singapore 637459, Singapore

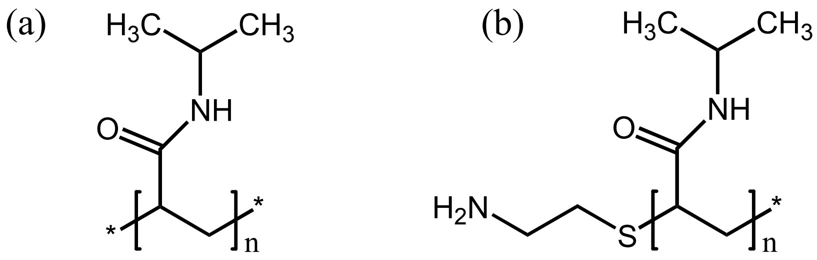

Fig. S1 Molecular structures of (a) PNIPAM and (b) PNIPAM-NH_2.

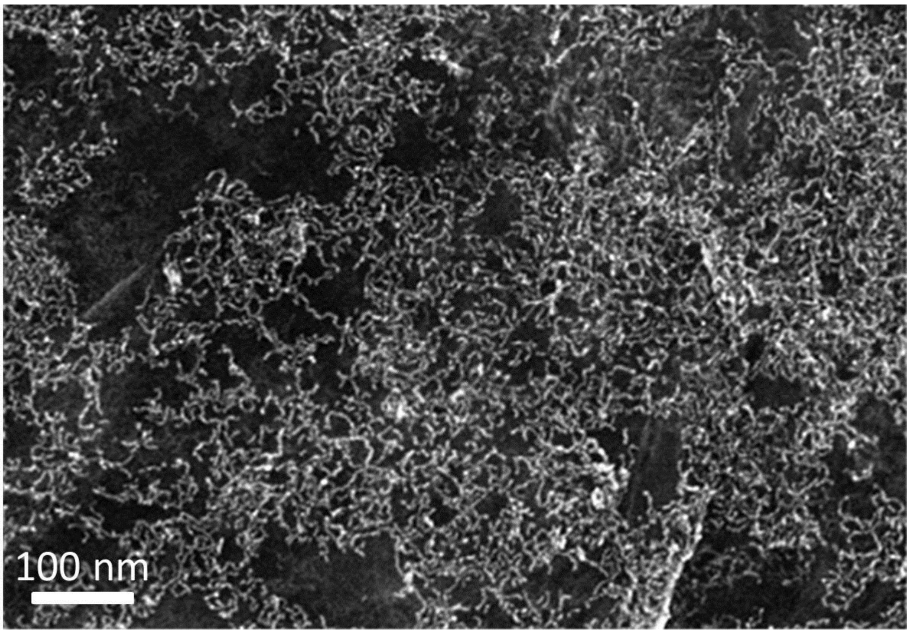

Fig. S2 SEM image of the $CuPt_3$ wavy nanowires.

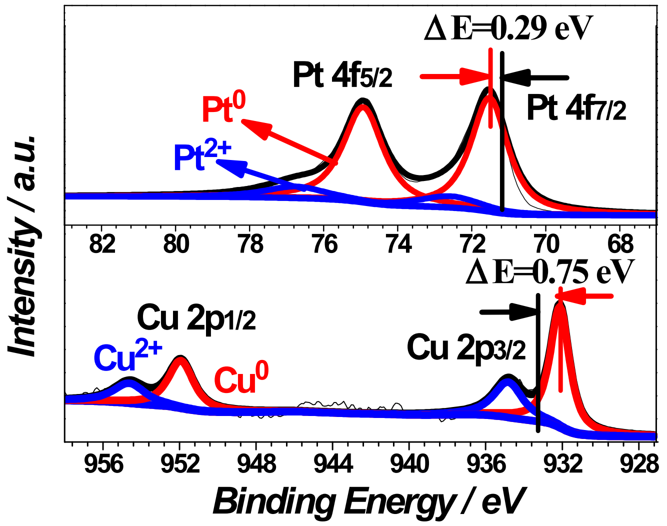

Fig. S3 High-resolution Pt 4f and Cu 2p XPS spectra of the $CuPt_3$ wavy nanowires. (Note: the vertical black lines represent the standard value of Pt 4f7/2 and Cu 2p3/2, respectively).

By measuring the relative peak areas, it is found that the percentages of Pt and Cu oxidation state at the $CuPt_3$ wavy nanowires remain at a low state (Pt^{2+}:15.2%; Cu^{2+}:32.0%).

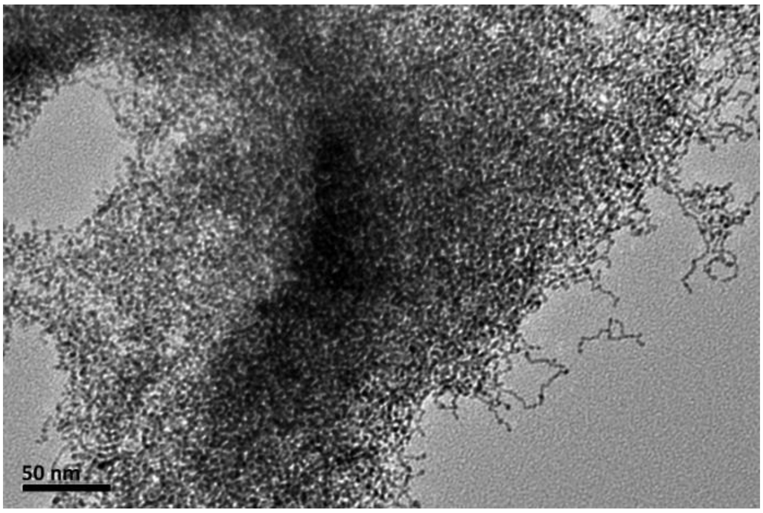

Fig. S4 TEM image of the $CuPt_3$ nanowires. (Note: The sample was prepared by placing a drop of the colloidal solution or catalyst powder dispersion in 50 ^{o}C ethanol solution (99%) on a carbon film coated Ni grid, followed by drying under 50 ^{o}C)

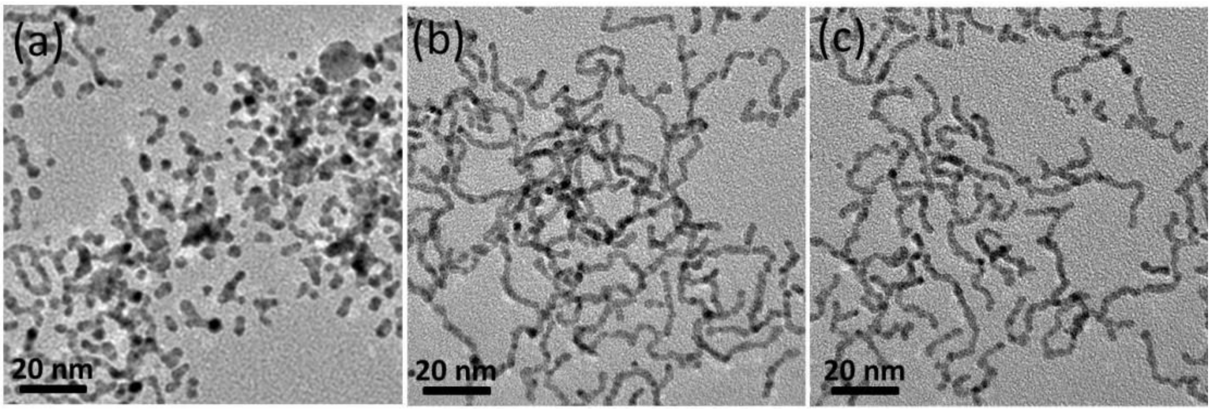

Fig. S5 TEM images of the $CuPt_3$ nanostructures prepared using the standard procedure, except for the use of different amount of PNIPAM-NH_2: (a) 0.5 ml, (b) 1.0 ml and (c) 2.0 ml.

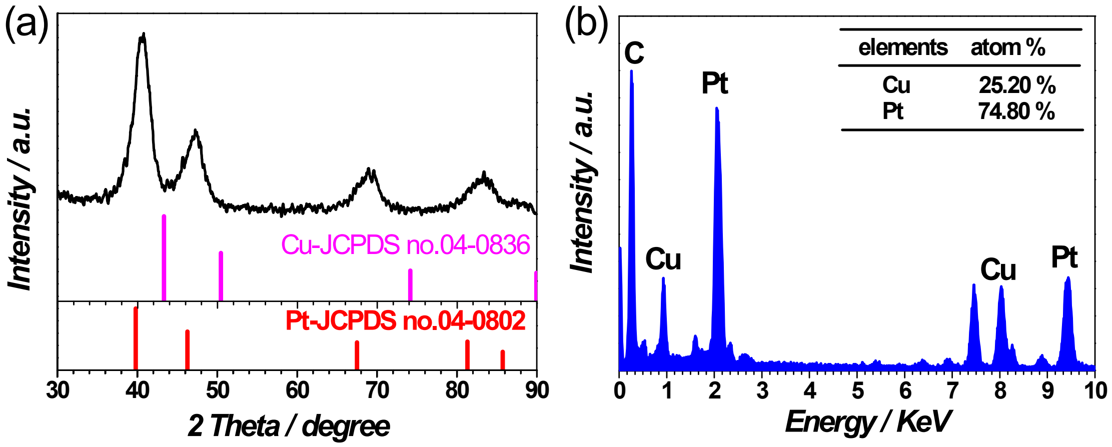

Fig. S6 (a) XRD pattern and (b) EDX spectrum of the products shown in Fig. S5a.

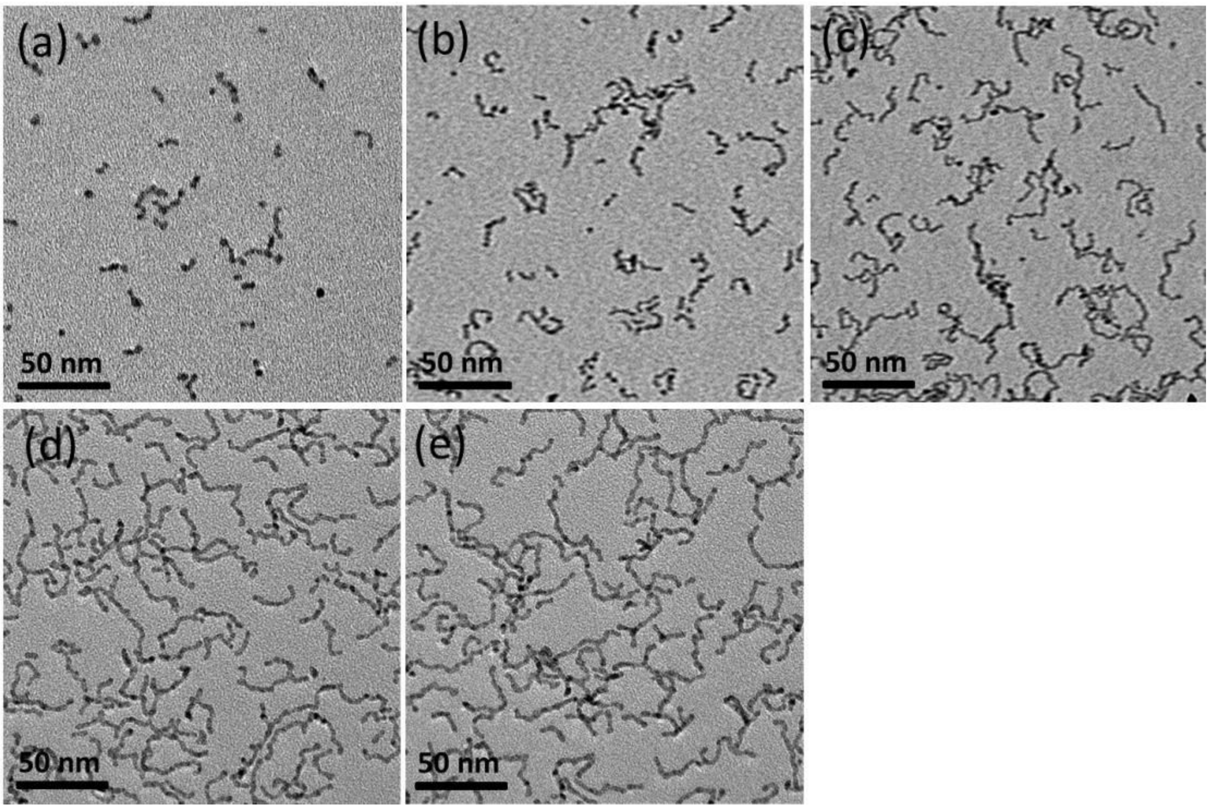

Fig. S7 Magnified TEM images of the $CuPt_3$ wavy nanowires collected at different growth stages: (a) 0.5 h, (b) 1 h, (c) 2 h, (d) 4 h and (e) 6 h.

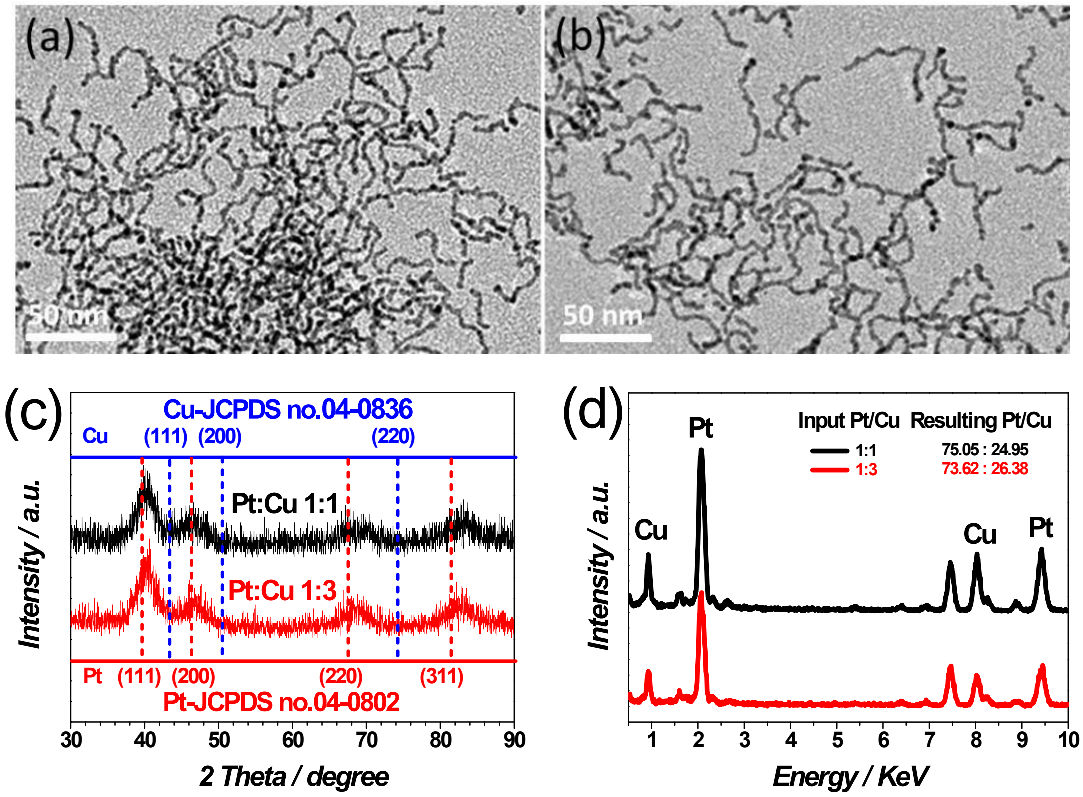

Fig. S8 TEM images of $CuPt_3$ wavy nanowires prepared with different input molar ratio of Cu/Pt: (a) 1:1 and (b) 3:1. (c) The corresponding XRD patterns and (d) EDX spectra for the $CuPt_3$ wavy nanowires prepared with different input molar ratio of Pt/Cu.

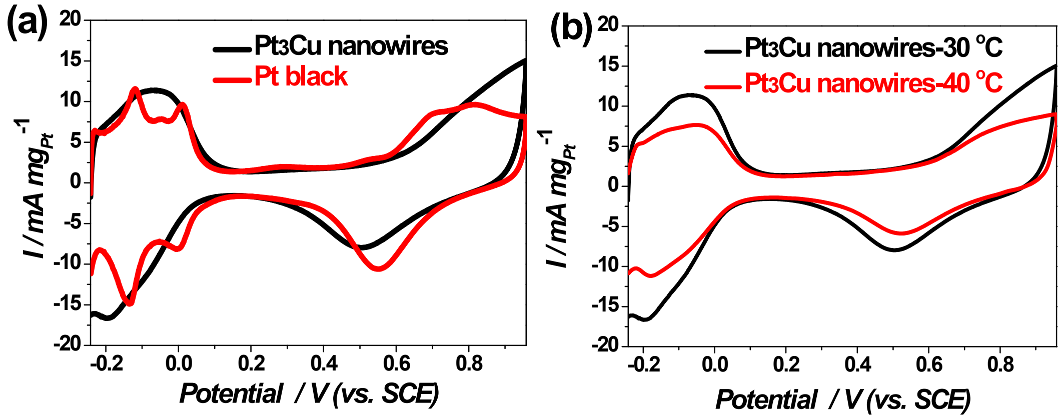

Fig. S9 (a) CVs for the $CuPt_3$ wavy nanowires and Pt black at 30°C in N_2-saturated 0.5 M H_2SO_4 solution at the scan rate of 50 mV s^{-1}. (b) CVs for the $CuPt_3$ wavy nanowires at two representative temperatures: 30 °C and 40 °C.

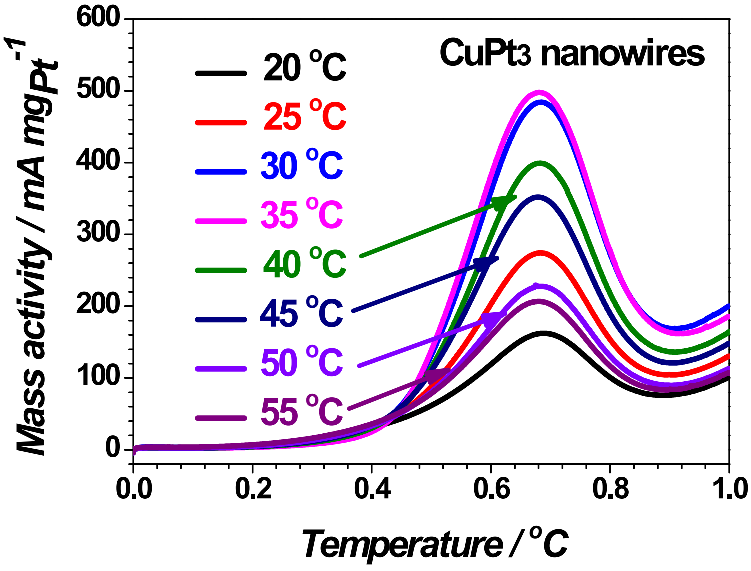

Fig. S10 CVs for the $CuPt_3$ wavy nanowires recorded at different temperature in N_2-saturated 0.5 M CH_3OH + 0.5 M H_2SO_4 solution at the scan rate of 50 mV s^{-1}.

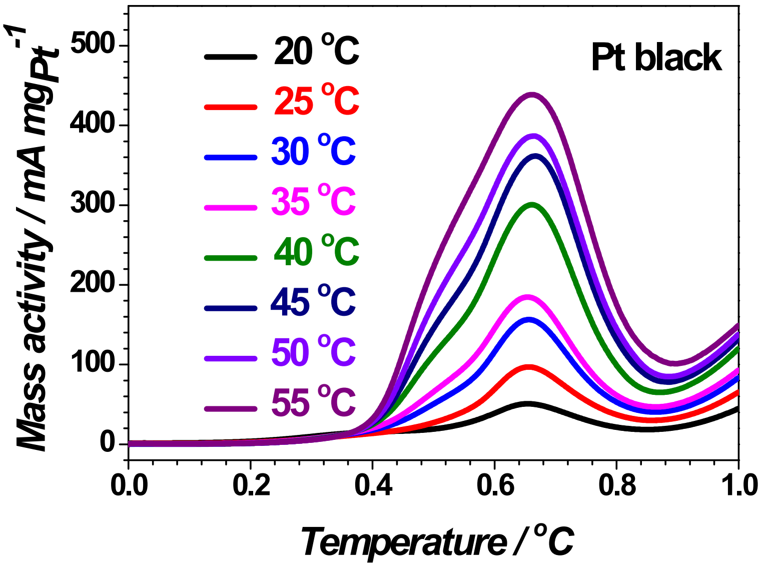

Fig. S11 CVs for the Pt black recorded at different temperature in N_2-saturated 0.5 M CH_3OH + 0.5 M H_2SO_4 solution at the scan rate of 50 mV s^{-1}.

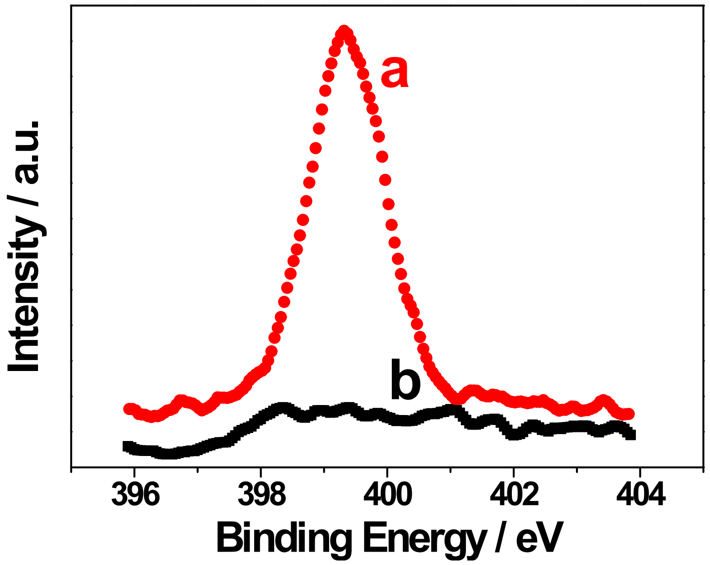

Fig. S12 XPS spectrum of the $CuPt_3$ nanowires in the N1s region (a) before and (b) after UV/Ozone treatment.

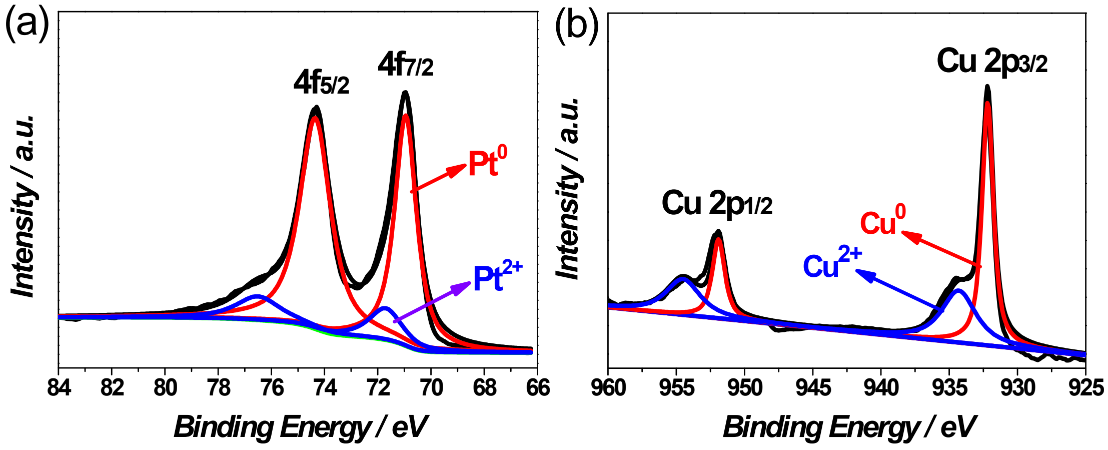

Fig. S13 High-resolution (a) Pt 4f and (b) Cu 2p XPS spectra of the “clean” CuPt3 wavy nanowires.

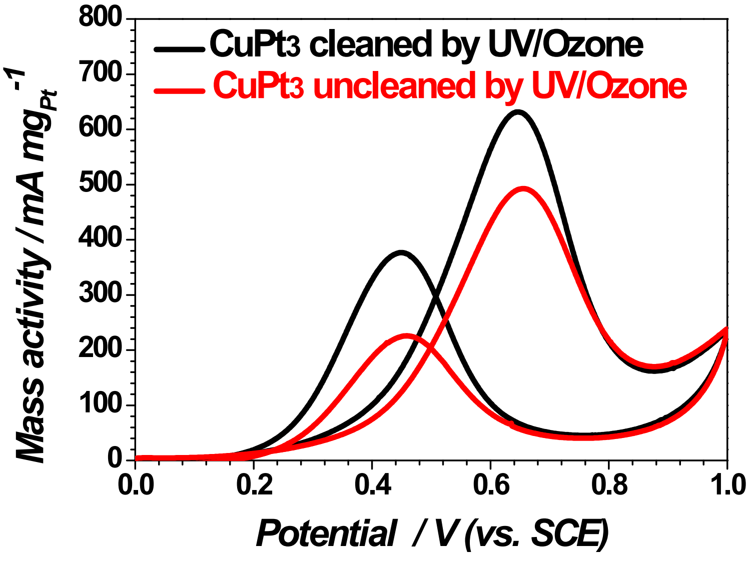

Fig. S14 Mass activities of the $CuPt_3$ nanowires before and after UV/Ozone treatment in N_2-saturated 0.5 M CH_3OH + 0.5 M H_2SO_4 solution at the scan rate of 50 mV s^{-1}.

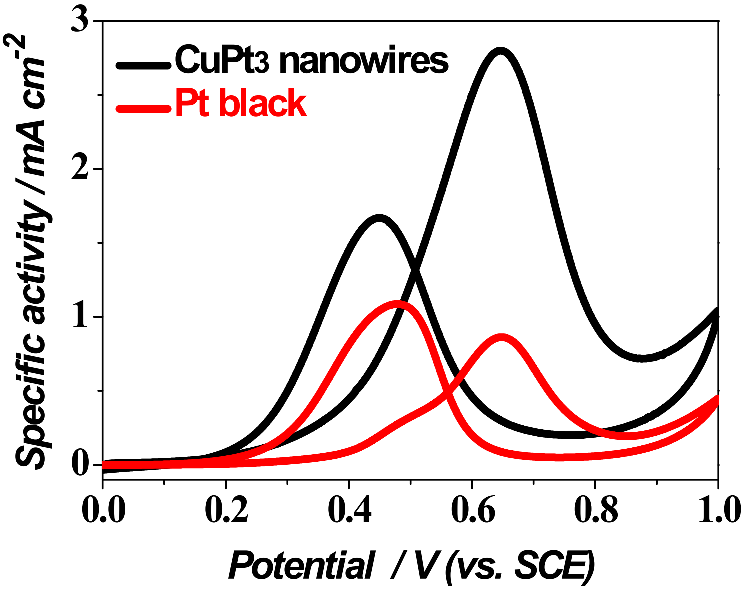

Fig. S15 Specific activities for the $CuPt_3$ nanowires and Pt black in N_2-saturated 0.5 M CH_3OH + 0.5 M H_2SO_4 solution at the scan rate of 50 mV s^{-1}.

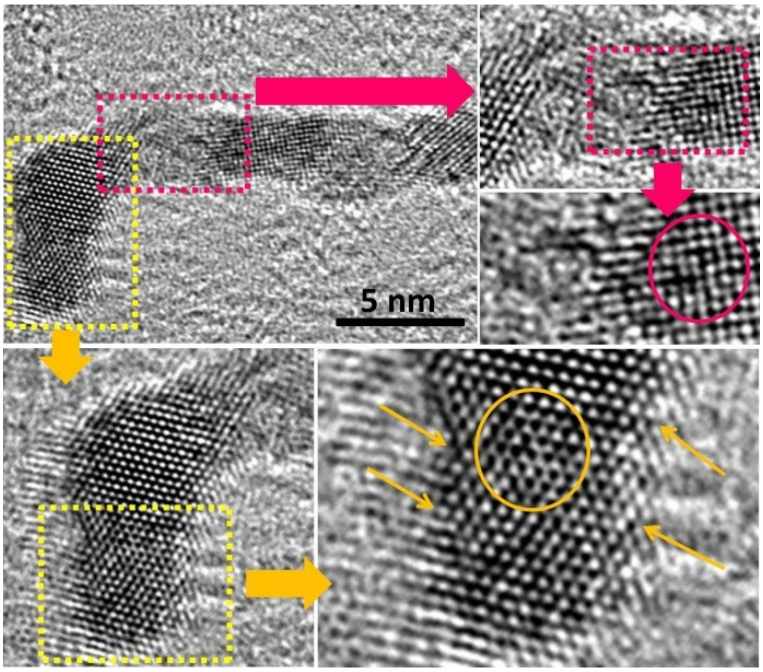

Fig. S16 High-resolution TEM images of $CuPt_3$ wavy nanowires at different regions. The atomic steps and crystal face defects exposed on the surface are indicated by arrows and cycles.
